# *Spirulina platensis* prevents oxidative stress and inflammation promoted by strength training in rats: dose-response relation study

**DOI:** 10.1038/s41598-020-63272-5

**Published:** 2020-04-14

**Authors:** Aline de Freitas Brito, Alexandre Sérgio Silva, Caio Victor Coutinho de Oliveira, Alesandra Araújo de Souza, Paula Benvindo Ferreira, Iara Leão Luna de Souza, Layanne Cabral da Cunha Araujo, Gustavo da Silva Félix, Renata de Souza Sampaio, Renata Leite Tavares, Reabias de Andrade Pereira, Manoel Miranda Neto, Bagnólia Araújo da Silva

**Affiliations:** 1 School of Physical Education, University of Pernambuco, Recife, Pernambuco Brazil; 2 Post-Graduation Program in Physical Education, University of Pernambuco/Federal University of Paraiba, Recife/João Pessoa, Pernambuco/Paraíba Brazil; 3 Physical Education Department, Health Sciences Center, Federal University of Paraiba, João Pessoa, Paraíba Brazil; 4 Laboratory of Studies of Physical Training Applied to the Performance and the Health/Health Sciences Center/Federal University of Paraiba, João Pessoa, Paraíba Brazil; 5 Medical Sciences Faculty, Facisa, Campina Grande, Paraíba Brazil; 6 Federal University of Tocantins, Licentiate in Physical Education, Tocantinopolis, Tocantins Brazil; 7 Postgraduate Program in Natural and Synthetic Products Bioactive/Health Sciences Center, Federal University of Paraiba, João Pessoa, Paraíba Brazil; 8 University of São Paulo, Institute of Biomedical Sciences, Department of Biophysics and Physiology, São Paulo, São Paulo Brazil; 9 Post-Graduation Program in Nutrition Science, Federal University of Paraiba, João Pessoa, Paraíba Brazil; 10 Pharmaceutical Sciences Department/Health Sciences Center/Federal University of Paraiba, João Pessoa, Paraíba Brazil; 11 Roraima State University, Department of Biological Sciences and Health, Boa Vista, Roraima Brazil

**Keywords:** Oxidoreductases, Prognostic markers

## Abstract

The purpose of this study was to evaluate the effects of *Spirulina Platensis* supplementation on selected blood markers of oxidative stress, muscle damage, inflammation, and performance in trained rats. Rats (250 g - 300 g) were submitted to a strength training program (eight weeks), divided into four groups: control (GT) (trained without supplementation), trained with daily-supplementation of 50 mg/kg (GT50), 150 mg/kg (GT150) and 500 mg/kg (GT500). Training consisted of a jump protocol in PVC-cylinder containing water, with increasing load over experimental weeks. We evaluated the markers of oxidative stress (malondialdehyde - MDA and antioxidant capacity) and inflammation (C-reactive protein) at the end of the training. Among groups submitted to strength training, concentration of C-reactive protein decreased after 8 weeks of intervention in the trained group and GT500. Strength training enhanced plasma MDA concentration of malondialdehyde with supplementation of S. platensis in GT150 and GT500. In plasma analysis, strength training enhanced the percentage of oxidation inhibition, with spirulina supplementation in rates of 150 and 500 mg/kg. Spirulina supplementation for 8 weeks (in a dose-effect manner) improved antioxidant capacity as well as attenuated exercise-induced increases in ROS and inflammation. As a practical application, the use as high doses did not cause a reduction in positive physiological adaptations to exercise training. Additional studies are necessary to test the application of *Spirulina Platensis* in other contexts, as collective sports (basketball, football, soccer).

## Introduction

*Spirulina platensis* is a microalga with biological activity as antioxidant, immunomodulatory, and anti-inflammatory and nowadays is used to produce nutritional supplements^1–3^. *S. platensis* is composed of protein (55%–70%)^4^, carbohydrates (15%–20%)^5^, lipids (approximately 7%)^5^, fiber, ash, and water including various minerals, vitamins, γ-linolenic acid, chlorophyll, carotenoids, and phycocyanin^2,6^. Recently, some researchers have reported that the latter played a crucial role in the antioxidative action of *S. platensis*^2^.

In this way, many animal and human studies have reported possible beneficial effects of *S. platensis* under several diseases such as diabetes^7^, dyslipidemia^8^, and chronic obstructive pulmonary diseases^9^. In most cases, the decrease in the oxidative stress and the inflammatory process reportedly had beneficial effects^2^.

In addition to clinical settings, *S. platensis* has demonstrated promising results in an exercise context^10^. In India, athletes have been eating *S. platensis* while training for track and field events^11^. Chinese and Cuban Olympic teams are also known to eat *S. platensis* daily during their training^12^. It suggests some strength or exercise performance related to the effects of *S. platensis* supplementation in humans^11^. Concomitantly, we know that high volume and intensity of physical training imposes a challenge for athletes to modulate their immune system, as well as promotes an increase of reactive oxygen species (ROS) production^11,13^, that are associated with early fatigue. Due to its nutritional, immunosuppressive, and antioxidant properties, *Spirulina platensis* can protect against early fatigue onset.

In recent years, several studies have reported ergogenic effects in athletes using raw or unprocessed foods^14^. Evidence suggests that many phytochemicals (usually not yet fully known) present in these foods can act synergistically, increasing the effectiveness of physiological outcomes, and decreasing possible adverse effects, such as toxicity, and positive adaptations to training^15^. The latter decreased with supplementation (vitamin C, E, and Resveratrol)^16–18^. Thus, we can infer that *S. platensis* (that contains several compounds with bioactive properties and different mechanisms of action) can promote ergogenic action in the context of physical exercise with minimized negative effects.

To date, only aerobic exercises were used to analyze *S. platensis* supplementation on markers of oxidative stress, inflammation, muscle damage, and performance^19,20^. Previous studies could not answer whether the ergogenic effect of *S. platensis* is replicable in strength exercises. Therefore, this study aimed to investigate *S. platensis* supplementation on oxidative stress and inflammation and whether possible improvements in these variables would result in better performance in trained rats.

## Methods

### Preparation and administration of *Spirulina platensis*

*Spirulina platensis* in powder form was obtained from Bio-Engineering Dongtai Top Co. Ltd laboratory (Nanjing, China) (Lot n°. 20130320). The extract was certificated by Pharma Nostra Quality Control Laboratory (Anápolis-GO, Brazil) (Lot No. 1308771 A); and preparing lyophilized by Dilecta Manipulation (João Pessoa-PB, Brazil) (lot n°. 20121025). The preparation of each dose of *S. platensis* was daily dissolved in saline in the proportions of 0.005, 0.015 and 0.05 g/mL for the preparation of the doses of 50, 150 and 500 mg/kg, respectively, which were administered to animals at the end of the preparation. We delivered the supplements over a period of eight weeks for all doses (50, 150 and 500 mg/kg/day) (BD-12, Insight, Ribeirão Preto, SP, Brazil) adapted from (Juarez-Oropeza *et al*., 2007)^21^. The oral administration was done from 12 to 14 hours, using stainless steel gavage needles (BD-12, Insight, Ribeirão Preto, SP) and 10 mL disposable syringes with 0.2 mL accuracy (BD, HIGILAB, João Pessoa, PB). At the beginning of each week, the animals in each group were weighed in order to consider the average weight of each group to properly calculate the gavage volume for each dose, considering the average animal weight. For groups undergoing strength training, supplementation was administered 30 minutes before the exercise session.

### Animals, ethical and experimental protocol

Wistar rats (Rattus norvegicus), weighing between 250 and 300 g, were obtained from Prof. Thomas George’s vivarium of the Center for Biotechnology (CBiotec/UFPB). The animals were kept under a balanced diet to feed base (Labina®) with free access to water. All experiments occurred from 08:00 h to 20:00 h, following guidelines for ethical animal use^22^, previously approved by the Ethics Committee of CBiotec (n° 0511/13). Forty animals were divided into groups subjected to the same strength training protocol and supplemented with *S. platensis* or saline (0.9% NaCl): trained group (GT; control group, n = 10), supplemented with saline, and trained group 50 (GT50, n = 10), (GT150, n = 10) and (GT500, n = 10), supplemented with *S. platensis*.

### Strength training program

Animals from the strength training group underwent a specific jump protocol in a PVC cylinder with 30 cm in diameter and 70 cm length, containing water. Water depth was 50 cm, close to twice the rat height, to avoid the animals doing alternative climbing and clinging to the cylinder’s edge, not to perform the exercises. The water was at a temperature of around 32 °C^23^. Based on Marqueti *et al*. (2006)^23^, the protocol of strength training through jumps in the water consists of progressively increasing the load as it adjusts to body weight. Overload was applied to the animals’ chest through a special vest that did not disconnect or restrict movement during jumps. Two-hour sessions, from 12:00 to 14:00 h, took place three times a week on alternate days. During each exercise series, the analysis of the time taken by the animal to perform the exercise evaluated the effectiveness of exercise on muscle performance.

Strength training and overload adjustments were developed as follows (Table 1):

1. *Adaptation Week -* performed with 50% of overload corresponding to animal body weight (1st day: 2 sets x 5 jumps; day 2: 4 sets x 5 jumps and day 3: 4 sets x 9 jumps) and a 30-second rest between sets
2. *1*^*st*^ *and 2*^*nd*^ *Weeks -* 4 set of 10 jumps with and an overload corresponding to 50% and a 30-second rest between sets
3. *3*^*rd*^ *and 4*^*th*^ *weeks -* 4 sets of 10 jumps, with a 30-second rest between sets and an overload corresponding to 60%.
4. *5*^*th*^ *and 6*^*th*^ *weeks -* 4 sets of 10 jumps, with an overload corresponding to 80% and a 30-second rest between sets.
5. *7th and 8th weeks -* 4 sets of 12 jumps with an overload of 80% and a 30-second rest between sets.

**Table 1:** experimental design of the strength training protocol.

Adaptation	Weeks			
	1^st^ and 2^nd^	3^rd^ and 4^th^	5^th^ and 6^th^	7^th^ and 8^th^
2–4 sets x 5–9 jumps (50%)	4 sets x 10 jumps (50%)	4 sets x 10 jumps (60%)	4 sets x 10 jumps (80%)	4 sets x 12 jumps (80%)

To evaluate exercise effectiveness on muscle strength, the runtime that the animal spent performing the same movement was assessed during each exercise. After 48 hours from the last training session and supplementing, animals were euthanized by cervical dislocation followed by cervical vessels section^24^.

### Biochemical measurements

After euthanasia of the animals, we collected 3 mL of blood through cardiac puncture^25^. From it, 1 mL of blood was collected immediately for determination of C-reactive protein (CRP)^26^. The other 2 mL was placed in test tubes containing (EDTA) to determine nitrite, malondialdehyde (MDA), and antioxidant activity^26^. Samples were centrifuged in a CENTRIBIO centrifuge, model 80-2B-15ML (Guarulhos- SP, Brazil). The supernatant or serum was transferred to Eppendorf tubes and refrigerated at -20 °C until analysis.

The CRP activities assay followed the method described by the International Federation of Clinical Chemistry and Laboratory Medicine^26^, using the commercial kit Labtest (Minas Gerais, Brazil). Absorbance was read in an automatic analyzer, LabMax 240 premium, at 340 nm for CK and LDH, and 540 nm for CRP, at ambient temperature.

### Assessment of malondialdehyde (MDA) levels

Samples of liver and quadriceps muscle were quickly removed, cleaned with Krebs solution to remove remaining blood, placed in Eppendorf tubes and stored in a freezer at -80 °C until analysis. After these tissues were homogenized with a 10% KCl ratio of 1:1, both 250 µL of plasma and homogenate were removed separately and incubated in a water bath at 37 °C for 60 minutes. Later, samples from both preparations were precipitated by Ohkawa *et al*.^27^. The material was read in a spectrophotometer (Biospectro, SP-220 model/Brazil) at 532 nm wavelength. A standard curve determined the MDA concentration in each plasma sample or tissue^10^. In tissues, absorbance values obtained were normalized by dry weight from the given sample volume.

### Evaluation of antioxidant activity

The basis of the procedure was the method described by Brand-Williams *et al*.^28^. The supernatant was necessary for the reading in a spectrophotometer at 515 nm (Biospectro, model SP-220/Brazil). The results were expressed as a percentage of oxidation inhibition, wherein: AOA = 100 − ([DPPH•R]T/[DPPH•R]B 100). Where [DPPH•R]T and [DPPH • R]B corresponding to DPPH concentration remaining after 30 minutes, measured in the sample (T) and blank (B) prepared with distilled water.

### Statistical analysis

Results were expressed as mean and standard deviation (SD). These results were analyzed statistically using analysis of variance (ANOVA) two-way followed by Bonferroni post-test, where the differences between means were considered significant when p ≤ 0.05. All results were analyzed by GraphPadPrism® program version 5.01 (GraphPad Software Inc., San Diego CA, USA).

## Results

### Effect of strength training and *S. platensis* supplementation on systemic inflammation level in rats

**Figure 1:**
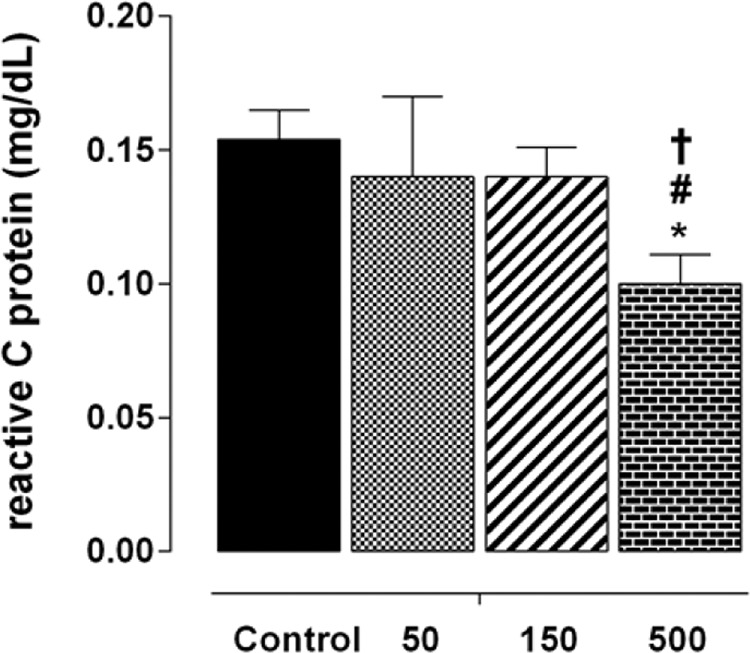
C-reactive protein after eight weeks of exercise for the groups () GT () and GT50 (), GT150 () and GT500 (). Vertical columns and bars represent mean and standard deviation, respectively (n = 8). “Two-way” ANOVA followed by Bonferroni post-test. *p ≤ 0.05 GT *vs*. GT500; ^#^*p* ≤ 0.05 GT50 *vs*. GT500; ^†^*p* ≤ 0.05 GT150 *vs*. GT500.

Among groups submitted to strength training, the concentration of C-reactive protein decreased after eight weeks of intervention in the trained group and supplemented with 500 mg/kg, but this decrease was significant when compared to the group submitted only to strength training (0.100 ± 0.011 *vs*. 0.154 ± 0.011 mg/dL, respectively). Trained group values supplemented with 50 mg/kg was pre (0.100 ± 0.011) *vs*. post 0.140 ± 0.03 mg/dL), and trained group values supplemented with 150 mg/kg was pre (0.100 ± 0.011) *vs*. post (0.140 ± 0.011 mg/dL). Thus, data reflect training the effectiveness as well as effectiveness of the most concentrated supplementation (Fig. 1).

### Effect of strength training and *Spirulina platensis* supplementation on the production of malondialdehyde

**Figure 2:**
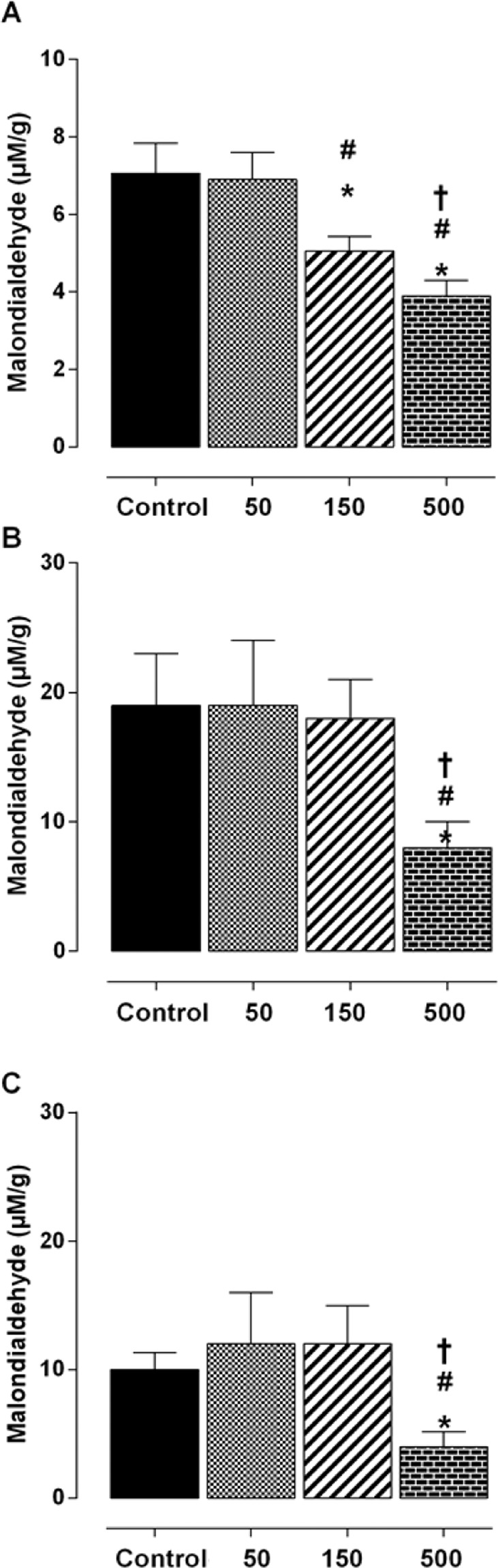
Malondialdehyde concentration in blood plasma (A), liver (B) and quadriceps (C) obtained after eight weeks of treatment groups GT(), GT50 (), GT150 () and GT500 (). Vertical columns and bars represent mean and standard deviation, respectively (n = 8). *p≤ GT vs. GT150; GT vs. GT500; ^#^p ≤ 0.05; GT50 vs. GT150; GT50 vs. GT500; ^†^*p* < 0.01; GT150 vs. GT500.

**Figure 3:**
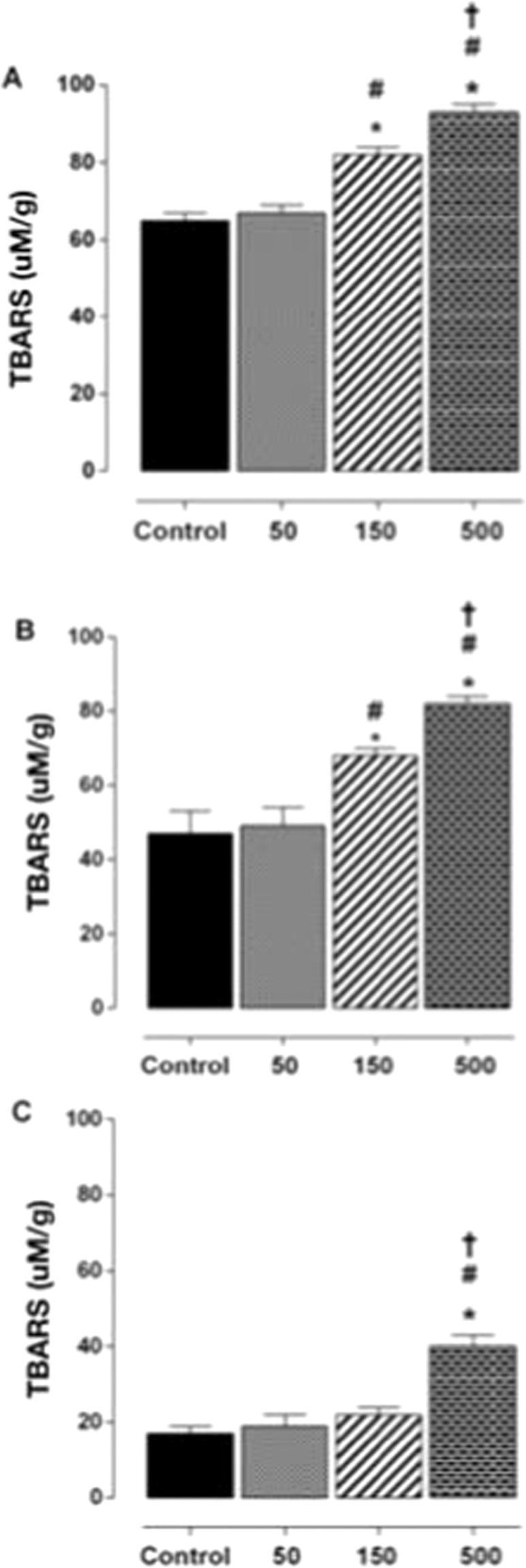
Antioxidant activity percentage in blood plasma (**A**), liver (**B**) and quadriceps (**C**) obtained after eight weeks of treatment GT () and GT50 (), GT150 () and GT500 (). Vertical columns and bars represent mean and standard deviation, respectively (n = 8).*p ≤ 0.05; GT vs. GT150; GT vs. GT500; ^#^p ≤ 0.05; GT50 vs. GT150; GT50 vs. GT500; ^†^*p* < 0.01; GT150 vs. GT500.

Strength training enhanced the decreased plasma MDA concentration of malondialdehyde with *S. platensis* supplementation at rates of 150 and 500 mg/kg. Values decreased from 7.1 ± 1.0 (GT50) and 6.9 ± 2.0 (GT50) to 5.6 ± 0.4 (GT150) and 3.9 ± 0.4 (GT500) nmol/L, yet less than GT500 and GT150 (Fig. 3A). Unlike plasma, the liver, when the trained group was supplemented with 500 mg/kg, presented lower MDA production compared to sedentary group exposed to the same condition (8 ± 2 vs 18 ± 2 uM/g) (Fig. 3B). Similarly, in the quadriceps, GT500 showed a lower MDA production regarding WG (4 ± 2 vs 10 ± 2 uM/g) (Fig. 2).

### Effect of strength training and *Spirulina platensis* supplementation in antioxidant activity determination

In plasma analysis, strength training enhanced the percentage of antioxidant activity with *S. platensis* supplementation in rates of 150 and 500 mg/kg. Values increased from 65 ± 2 (GT) and 67 ± 2 (GT50) to 82 ± 2 (GT150) and 93 ± 2 (GT500), though greater than GT500 and GT150. In the liver, the trained group supplemented with 150 mg/kg (68 ± 2%) and 500 mg/kg (78 ± 3%) showed a higher percentage of antioxidant activity compared to GT and GT50 (45 ± 2; 50 ± 2 uM/g). And in the quadriceps muscle, GT500 had a higher antioxidant activity percentage compared to GT, GT150, and GT150 (40 ± 3 *vs*. 18 ± 2, 20 ± 3 and 23 ± 2%) (Fig. 3).

## Discussion

To our knowledge, this is the first study to examine the effects of chronic *Spirulina* supplementation on markers of oxidative stress and inflammation related to resistance training. Results showed that *Spirulina* supplementation for eight weeks (in a dose-effect manner) induced a significant improvement in the antioxidant capacity (AC) as well as attenuated exercise-induced increases in ROS (measured by MDA) and inflammation (CRP). Our research group recently demonstrated that *Spirulina* supplementation for eight weeks induced significant improvement in exercise performance (time of execution) and decrease of muscle damage with the reduction of creatine kinase (CK) and lactated dehydrogenase (LDH) (Brito *et al*.)^10^.

The combination of phytochemicals can exhibit complementary and overlapping mechanisms of action, including antioxidant activity, and scavenging free radicals; modulation of enzymes activity in detoxification, oxidation, and reduction; and stimulation of the immune system and regulation of hormone metabolism^29,30^. Besides, undesirable effects such as induction of pro-oxidant states and decrease of mitochondrial biogenesis and myofibrillar hypertrophy have been recently demonstrated with dietary supplementation (isolated: vitamins C, E, and Resveratrol)^31–34^. By presenting various phytochemicals, especially phycocyanin, *S. platensis* shows great potential for its employment in sports, with possible deleterious effects minimized.

Dose analysis of dietary intervention is of most importance. In this context, only^35^ attempted to identify *S. platensis* dose-response. Our study confirms their findings since we observed that 500 mg/kg showed as more effective. Adverse effects have been observed with high doses of fish oil - inflammatory aspects^36^ and *bulbine natalensis* – hormonal aspects^37^. Thus, it appears that the use of *S. platensis* is a viable supplementation alternative regarding adverse events (with high doses). Further dose–response studies are required.

Supplements antioxidants may benefit exercise performance, directly by a muscle fatigue decrease and indirectly by a decrease of physiological stressors or improvement to recover from training^33^. Results found in human studies conducted on *S. platensis* chronic consumption demonstrated there were moderate performance improvements in quadriceps 1 and in one rat study^34^. It is necessary to recognize that these studies were carried out with aerobic exercises, while our study contemplated strength training protocol. Thus, our data demonstrate, in an unprecedented manner (as shown by execution time), that *S. platensis* supplementation may benefit strength athletes (Brito *et al*.)^10^.

Regarding muscle damage, our study showed that higher doses (500 mg/kg) decreased CK and LDH. Similarly, Lu *et al*^38^. demonstrated positive changes in plasma concentrations of MDA, SOD, LDH, and GPx after Spirulina supplementation for three weeks in untrained individuals and a single exercise session. On the other hand, Franca *et al*^19^. did not show any improvements in muscle damage biomarkers (in well trained cyclists). About that, some considerations are mandatory. Biomarkers are frequently thought in terms of muscle damage. However, they differ in time, course of appearance, and disappearance after exercise. So, a study design concerning the timeline for blood sampling must take into account the exercise type, duration, and intensity, diet, and if the subjects recruited are trained or untrained^32^.

The parameters evaluated in our study were inflammation and oxidative stress. In this context, the investigations showed that systemic inflammation could be one of the physiological stress markers in athletes^12,39^ because this process is required to promote training adaptations. Our study shows that both parameters improved with *S. platensis* supplementation. Therefore, other markers as cytokines and CRP were used as inflammatory markers for manuscripts previously to us. Although CRP has a fast response (1 to 2 days), it remains increased for short periods (5 to 6 days)^40^. In our study, we evaluated CRP and saw a reduction in this inflammatory marker. Regarding oxidative stress, we evaluated lipid peroxidation markers, the thiobarbituric acid reactive substances (TBARS). We observed a decrease in them, demonstrating an improvement in oxidative stress. That observation suggests that supplementation with Spirulina in athletes can promote a decrease in systemic inflammation.

Although not regarded in this study, the physiological alterations and mechanisms by which Spirulina promotes performance improvement need to be further investigated due to the lack of published studies. Some other possible mechanisms included: increased supply of cysteine (cysteine is generally the limiting amino acid for GSHx synthesis in humans and other animals), inhibition of NF-Kb, COX-2, and NADPH oxidase^41–43^. Possible mechanism of *S. platensis* in the improvement of oxidative stress has been demonstrated by McCarty 2007^43^, where he showed that *S. platensis* inhibits NADPH oxidase, which is an enzyme that catalyzes the production of the superoxide free radical. Meanwhile, the reduction in the inflammatory process, as demonstrated in our study, was also seen in other studies, they showed that *S. platensis* reduces the production of proinflammatory cytokines such as TNF-α, IL-1β, and IL-6 by NF-κB pathway inhibition^42,43^.

### Conclusions section

This study is the first to demonstrate the use of *Spirulina platensis* in the context of physical training (high-intensity exercise) since it is effective in improving the performance and reducing muscle damage, oxidative stress, and inflammation. The chronic nature of the study (8 weeks) differs from other studies in the literature that only verified the acute responses to an exercise session. Therefore, proving it to be a dietary strategy of high applicability in sport. It is noteworthy that the use of high doses did not cause a reduction of positive physiological adaptations to exercise training, a fact already seen with some supplements (vitamins C, E, and Resveratrol). Additional studies are necessary to test the application of *Spirulina platensis* in other contexts, such as collective sports (such as basketball, football, and soccer) and during low-calorie diets (bodybuilding athletes, martial arts, and gymnastics).
